# Design and Validation of a Fiber-Reinforced Polymer Cable-Stayed Pedestrian Bridge: Human-Induced Actions vs. Comfort Levels

**DOI:** 10.3390/ma17122842

**Published:** 2024-06-11

**Authors:** Izabela Joanna Drygala, Nicola Nisticò

**Affiliations:** 1 Faculty of Civil Engineering, Cracow University of Technology, 31-155 Cracow, Poland; izabela.drygala@pk.edu.pl; 2 Department of Structural and Geotechnical Engineering, Sapienza University of Rome, 00184 Rome, Italy

**Keywords:** pedestrian bridge, FRP material, numerical modelling, dynamic analysis, vibration comfort criteria assessment

## Abstract

The investigation into advanced structural materials, such as composite materials, has revealed numerous possibilities within the field of bridge engineering. Glass-fiber-reinforced polymers (GFRPs) are notable among these materials, particularly in footbridge construction, encompassing both arch and cable-stayed designs. While GFRPs boast advantages, such as their high strength-to-weight ratio, they may exhibit some deficiencies, particularly when subjected to dynamic loads induced by wind or pedestrian forces. Two noteworthy global examples are the Lleida arch bridge (Spain, 2001) and the Aberfeldy cable-stayed bridge (Scotland, 1992). These structures have recently undergone comprehensive studies by the authors to assess their behavior when subjected to specific conditions regarding pedestrian traffic and vibrations induced by under-passing trains, as far as Lleida is concerned. The methodologies employed in these studies are detailed herein, incorporating the relevant scientific literature and technical regulations that provide guidance on fundamental principles for bridge design, pedestrian modelling, and acceleration thresholds aimed at minimizing discomfort. While the framework of principles is clear, the regulations are extensive, requiring designers to have a comprehensive understanding of the diverse outcomes achievable through various approaches. Therefore, the provided state-of-the-art overview serves as a roadmap for assessing the performance of an innovative cable-stayed bridge recently proposed by one of the authors. Initially designed with six spans, this prototype has been reconfigured here as a three-span train station overpass. The analyses conducted allowed for the assessment of induced accelerations. According to current accredited standards, the resulting comfort classification is considered minimal, even if, for crowded conditions, more specific studies are required.

## 1. Introduction

FRP bridges have been implemented worldwide for both vehicular and pedestrian purposes. Alper et al. (1977) [1], mentioning a bridge erected in Tel-Aviv (Israel) as forerunner of FRP applications, presented a reinforced plastic pedestrian bridge. Later, some, not many, bridges have been installed, with a comprehensive overview up to 2010 provided in [2], and a more recent review in [3] discussing solutions for an innovative FRP pedestrian cable-stayed bridge. Various typologies have been proposed, and innovative applications encompass the following: (1) cable-stayed and suspension typologies, specifically the Aberfeldy [4], Kolding [5] and the Wilcott [6,7]; (2) the Lleida [8] arch bridge; and (3) the truss Prato [9,10] and the U- shaped l [11,12]. Common challenges pertain to joint conception, durability, and the evaluation of the impact of actions induced by pedestrians and vehicular traffic.

Concerning pedestrian-induced vibrations, the design process can be supported by international recommendations [13,14,15,16], which (1) establish thresholds for vertical and lateral frequencies to avoid specific studies, (2) propose different risk levels based on peak induced accelerations, and (3) provide guidelines to model pedestrian actions discussed in the scientific literature, including studies reported in [17,18,19,20,21,22]. While these challenges are shared with traditional pedestrian bridges [23,24,25,26], the use of FRP does not eliminate them, even if critical damping [27] for FRP bridges surpass those of steel, concrete, and steel-composite footbridges.

In this context, the authors collaborated on a study [28], utilizing numerical simulations to assess the performance of the pioneering Aberfeldy FRP bridges under the effects of walking and running pedestrians. Building on the research by Drygala et al. (2019) [29], this study will be expanded to evaluate the performance of a variant of the Salerno cable-stayed bridge proposed by Nisticò and co-workers, as discussed in [3].

Thus, this paper will delve into the methodology adopted for evaluating pedestrian bridge performance and, as far as the Salerno variant is concerned, the assessment of the comfort against induced pedestrian and vehicular vibrations.

## 2. Methods and Standards

When assessing an existing bridge, a straightforward methodology involves the direct evaluation of comfort through experimental assessments. Groups of pedestrians can traverse the bridge along predefined paths at varying velocities. Given the diversity of pedestrian perceptions, it is essential to carefully select the pedestrian typology. The paths should be designed to encompass the most challenging conditions.

Each pedestrian, including those at rest, will provide their judgment on discomfort, which depends, among other factors, on pedestrian velocity. Discomfort tends to increase as velocity decreases. By monitoring the bridge decks using accelerometers, a relationship can be established between comfort levels and induced acceleration, denoted as a(t), over time (t).

Simultaneously, alongside experimental tests, numerical studies can be conducted to predict pedestrian-induced acceleration. Typically, these studies are performed for designing new bridges or developing specific systems (e.g., tuned mass dampers) to mitigate acceleration as necessary. It is noteworthy that Eurocode [14] explicitly acknowledges the inherent uncertainties in the data used for calculations, stating, ‘the results are subjected to very high uncertainty’. When comfort criteria are not adequately met, provisions may need to be made for post-completion installation of dampers in the structure. Within this framework, critical aspects, discussed in the following, regard pedestrian- and train-induced actions and comfort levels definition.

### 2.1. Pedestrian- and Train-Induced Actions

Bachman and his co-authors emerge as significant contributors in the investigation of vibration issues within structures. Their notable contributions, as documented in [17,18], are compiled in [25]. The spectrum of pedestrian activities, encompassing walking, jogging, and running, can be systematically categorized, and their resulting effects can be quantified through mathematical representations such as Fourier series. The classification depends on the velocity type of motion, frequency ($f_{u}$), velocity (v), and stride length ($l_{u}$) can be fitted with reference to Table 1.

The force evolution in the time can be approximated through harmonic series, as expressed in Equation (1), where (1) G—the pedestrian weight (assumed to be equal for each harmonic); (2) $\varphi_{n}$—the phase angle of the n-th harmonic; (3) $\alpha_{n}$—the dynamic load factor (DLF); and (4) $f_{n}$—the step frequency that for horizontal (lateral) action can be assumed to be half of the vertical frequency, as indicated in (SÉTRA [13]). The values of these parameters (G, $\varphi_{n}{,\alpha }_{n}$) have been proposed in the past, relying on experimental results. Suggested values, for both vertical and lateral directions, are detailed in Table 2, encompassing both walking and running activities.

(1) $$F\left(t\right)=G+G\sum_{n=1}^{k}a_{n}sin(2\pi nft+\varphi_{n})$$

It is worth noting that concerning running, the single foot action can be more accurately approximated by the half-sine expression [13], presented in Equation (2), where $G_{0}=0.7\;\mathrm{kN}$; t—time; j=1,2,…,n; T—step period (1/f); $k=t_{c}/T$—contact time factor; $t_{c}$—face time; and $A_{r}$—dynamic impact factor ($A_{r}=\pi /\left(2\cdot k\right)$ [20,25]. Typical values of $t_{c}$_,_ T, k, and $A_{r}$ are reported in Table 2.

(2) $$F\left(t\right)=\left\{\begin{array}{ccc} A_{r}G\sin\left(\frac{\pi f}{k}t\right) & for & \left(j-1\right)\cdot T\leq t\leq \;\left(j-1\right)\cdot T+t_{c}\\ 0 & for & \left(j-1\right)\cdot T+t_{c}<t\leq j\cdot T \end{array}\right.$$

The velocity and the resulting induced forces depend on pedestrian density, as detailed in [26]. In the instance of a low-density pedestrian stream (less than 0.6 [person/m^2^]), each pedestrian retains their individual velocity and step frequency. With escalating density, velocity diminishes, leading pedestrians to synchronize with each other. Notably, as they sense the vibrations of the bridge, they laterally move in resonance with the bridge itself.

Single pedestrian models can be adopted to simulate groups of walkers and runners. These models can be tuned differently in terms of time and space. Additionally, considering a continuous flow of N synchronized pedestrians, the following conditions apply: (1) they travel contemporarily with a constant velocity (V) along the deck with a length of L, and (2) the value of N is equal to Q×T, where Q represents the flow (number of persons per second) and T is defined as L/V, representing the time needed by pedestrians to traverse the entire length (L). In the case where pedestrians are not synchronized, an equivalent number of pedestrians (NEQ) can be defined to evaluate the equivalent action resulting from a phase shift.

**Figure 1:**
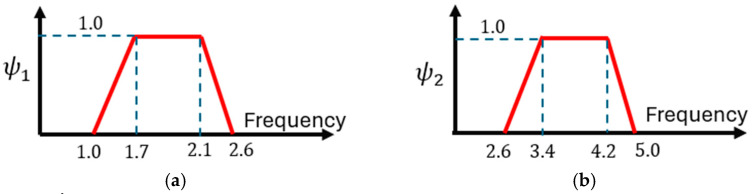
SÉTRA [13]. Crowd condition: values of the $\psi_{1,2}$ function (Equations (6) and (7)). (**a**) sparse and dense crowds; (**b**) effect of the second harmonic of the crowd.

In SÉTRA [13], Backman and the half-sine function are adopted, and two expressions are proposed to evaluate N_EQ_ in the case of a very dense crowd (Equation (3)) and a sparse and dense crowd (Equation (4)), introducing dependence on the structural critical damping (ξ). These expressions, derived from 500 experimental tests, represent the 95% characteristic value. It is worth noting that the number of pedestrians can be evaluated as reported in Equation (5), where A is the deck area, and ρ is the number of pedestrians per unit surface. The values for ρ can be assumed as spare (ρ=0.5), dense (ρ=0.8), and very dense (ρ=1.0). The resulting expression is reported in Equation (6), where $\psi_{1}$ is the frequency dependent function reported in Figure 1a. Further on, the second harmonic of the crowd needs to be considered in case of class I and II urban footbridges for which a very dense or dense condition is expected, respectively, and (1) a lower acting force needs to be considered, as reported in Equation (7), adopting (2) the function $\psi_{2}$ reported in Figure 1b.

(3) $$N_{EQ}=1.85\sqrt{N}$$

(4) $$N_{EQ}=10.8\sqrt{N\cdot \xi }$$

(5) N=A×ρ

(6) $$F\left(t\right)=\left[0.28\;\left(\mathrm{kN}\right)\cdot \psi_{1}\cdot cos(2\pi f_{1}t)\right]\cdot N_{EQ}$$

(7) $$F\left(t\right)=\left[0.07\;\left(\mathrm{kN}\right)\cdot \psi_{2}\cdot cos(2\pi f_{1}t)\right]\cdot N_{EQ}$$

**Figure 2:**
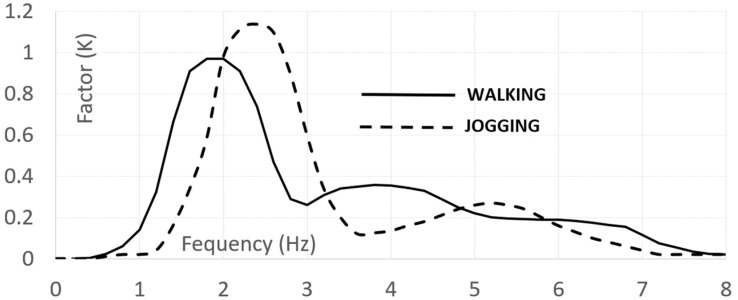
British Standard [16]. Value of $k\left(f_{v}\right)$.

**Figure 3:**
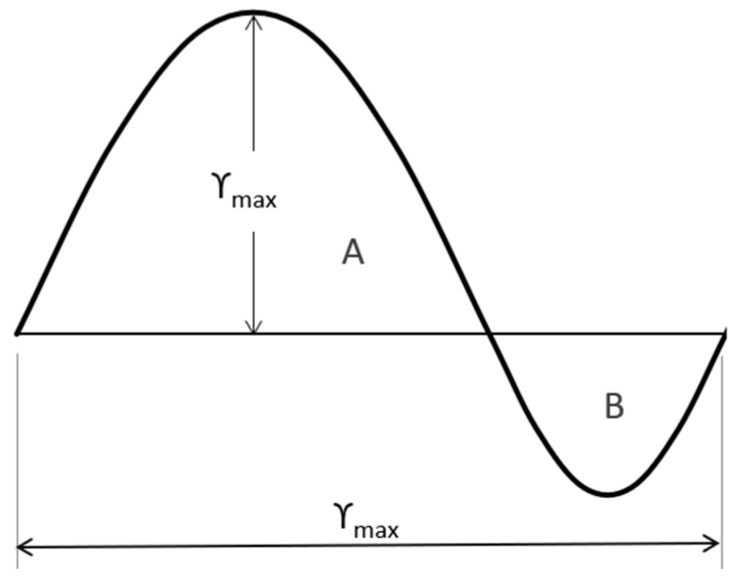
British Standard [16]. Value of $\lambda =0.634\frac{S}{S_{eff}}$, $S_{eff}=\frac{areA+AreB}{0.634\cdot \gamma_{max}}$.

**Figure 4:**
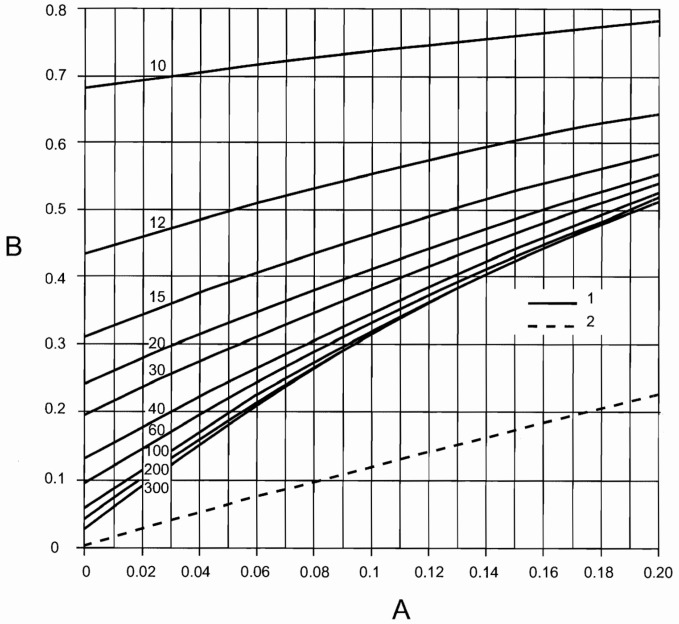
British Standard [16]. Reduction factor (γ, (B)) as function of structural damping (A). Solid lines (line no. 1) represent pedestrian groups, while the dashed line (line no. 2) indicates areas dedicated for crowd loading. Each solid line (line no. 1) is related to the effective span length [m] of the bridge.

The British Standard [16] is noteworthy among the codes incorporating models for pedestrian groups. Actions are represented by Equation (1), albeit with distinctions: the phase angle is omitted, only one harmonic is considered, and a factor is introduced to encompass unsynchronized combinations. For walking and running either in normal or crowded conditions, two expressions are given, as reported in Equations (8) and (9), that depend on a set of parameters that are as follows: $F_{0}$—amplitude of the pedestrian transmitted force; $f_{v}$—frequency of the force set to be equal to the most demanding bridge frequency; $k\left(f_{v}\right)$—dependent on frequency (see Figure 2) consider pedestrian sensitivity; N—total number of pedestrian with its value (see Table 3) contingent on usage; λ—reduction factor (see Figure 3) to account for a lower number of pedestrian, in crowd condition, when the mode of interest include internal nodes; and γ—reduction factor considering unsynchronized combinations of pedestrian actions, dependent on structural damping and structural span (Figure 4).

(8) $$F(t)=F_{0}\cdot k\left(f_{v}\right)\cdot \sqrt{1+\gamma \cdot \left(N-1\right)}\cdot sin\left(2\pi \cdot f\cdot t\right)$$

(9) w=1.8F0A·kfv·γ·Nλ·sin2π·f·t

To the best of the author’s knowledge, there is currently no dedicated literature addressing the quantification of pedestrian comfort during the passage of trains or vehicles in underpasses. The existing literature predominantly focuses on the impact of such factors on individuals within built environments. Notably, within the broader context of international standards, a comprehensive review is presented in [30,31,32,33,34,35,36].

**Figure 5:**
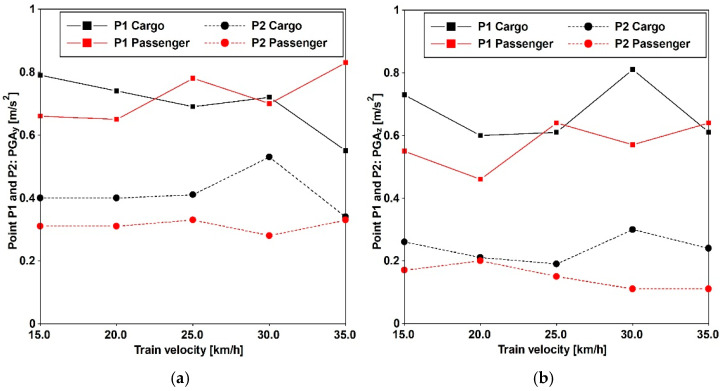
PGAs of the acquired acceleration histories, used for numerical simulation purposes regarding train-induced vibration, vary depending on the type of train (passenger and cargo) and its velocity. Horizontal (**a**) and vertical (**b**) direction.

**Figure 6:**
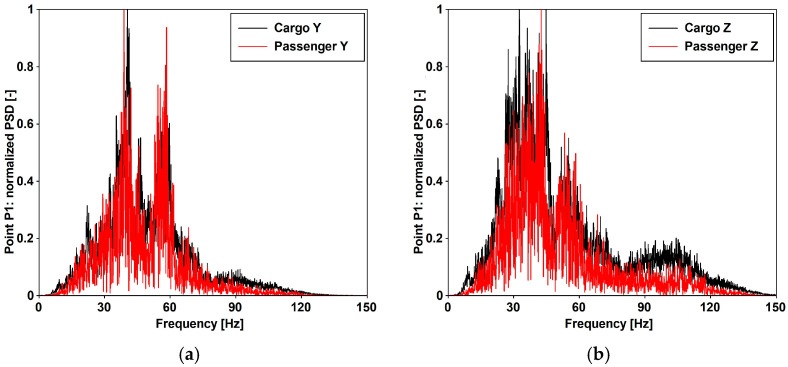
A comparison of the frequency domain characteristics for cargo and passenger trains with a velocity of 30 [km/h] for: (**a**) horizontal and (**b**) vertical direction.

Additionally, the impact of both cargo and passenger trains operating at various velocities is detailed in [29], utilizing data gathered at three specified positions differing in distance from the considered train position. Figure 5 and Table 4 provide the peak ground accelerations (PGAs) extracted from the acceleration time histories recorded at distances of 10 m (point P1) and 15 m (point P2). The frequency domain content of cargo and passenger trains traveling at a speed of 30 [km/h] is provided in Figure 6.

### 2.2. Comfort Levels, Related Accelerations, and Design

SÉTRA [13], Eurocode [14], and the British Standard [16] define the PAV threshold differently, considering it as a parameter crucial for ensuring an acceptable comfort level. It is important to acknowledge that these thresholds serve as reference values [26], and adherence to them does not eliminate the possibility of resonance risk.

According to SÉTRA [13], distinct ranges must be considered for PAV, as detailed in Table 5. Further, the levels of resonance risk are defined based on a series of thresholds (refer to Table 6).

According to Eurocode [14], the acceptable values are as follows: (1) $0.7\left[\frac{m}{s^{2}}\right]$ for the vertical direction; (2) 0.2 $\left[\frac{m}{s^{2}}\right]$ for horizontal vibrations due to normal use and for exceptional crowd conditions. The British Standard [16] establishes vertical lower and upper bound limits at $0.5\left[\frac{m}{s^{2}}\right]$ and $2.0\left[\frac{m}{s^{2}}\right]$, respectively. It recommends evaluating the PAV according to Equation (10), where the value of $k_{4}$ (exposure factor) can be equal to 1. Additionally, as outlined in Table 7, the other factor depends on site usage ($k_{1}$), redundancy ($k_{2}$), and structure height ($k_{3}$).

(10) $$a_{lim}=1.0\cdot k_{1}\cdot k_{2}\cdot k_{3}\cdot k_{4}\;\;\left[\frac{m}{s^{2}}\right]$$

**Figure 7:**
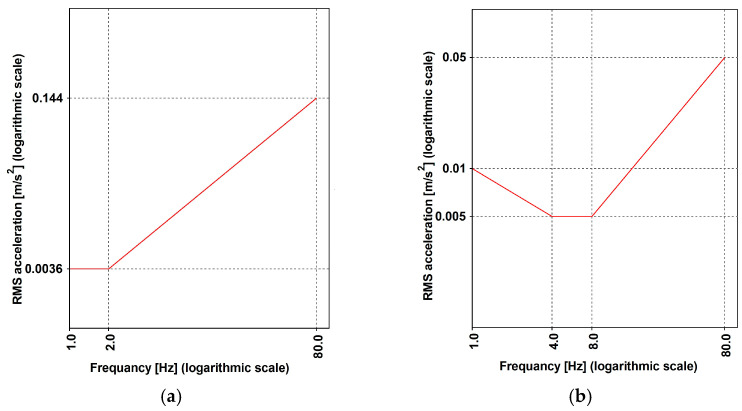
ISO [15]: RMS normalized thresholds for horizontal (**a**) and vertical (**b**) direction.

According to ISO [15], the root mean square (RMS) is chosen, according to Equation (11), as parameter, assuming ∆t=1.0 [s]. Thresholds are defined based on the frequency and acceleration direction, using a reference system local to the human body. The considered directions are horizontal and vertical, being further classified as side-to-side, back-to-chest, and foot-to-head. Pedestrian activity is classified as walking or standing depending on the velocity. The recommended normalized RMS values are reported in Figure 7. These values need to be appropriately scaled using the factors reported in Table 8.

(11) $$RMS={\left[\frac{1}{∆t}\int_{t_{1}}^{t_{1}+∆t}{\left[a\left(t\right)\right]}^{2}dt\right]}^{\frac{1}{2}}$$

The previous synthesis, referring to the frequency values reported in Table 9, outlines that the Prato truss bridge and the U-shaped Blackpool bridge adhere to the Euro Codes [17], which permit the omission of specific studies when vertical and horizontal frequencies surpass 5.0 and 2.5 [Hz], respectively. The Kolding structure is close to the recommended threshold. Furthermore, when considering the defined threshold in SÉTRA [13], the risk of resonance is as follows: (1) Aberfeldy has a medium and maximum risk for vertical and horizontal frequencies, respectively; (2) Lleida has a low risk; and (3) Wilcott has a maximum risk.

## 3. The Salerno Bridge: Prototype GFRP Footbridge as a Case Study

**Figure 8:**
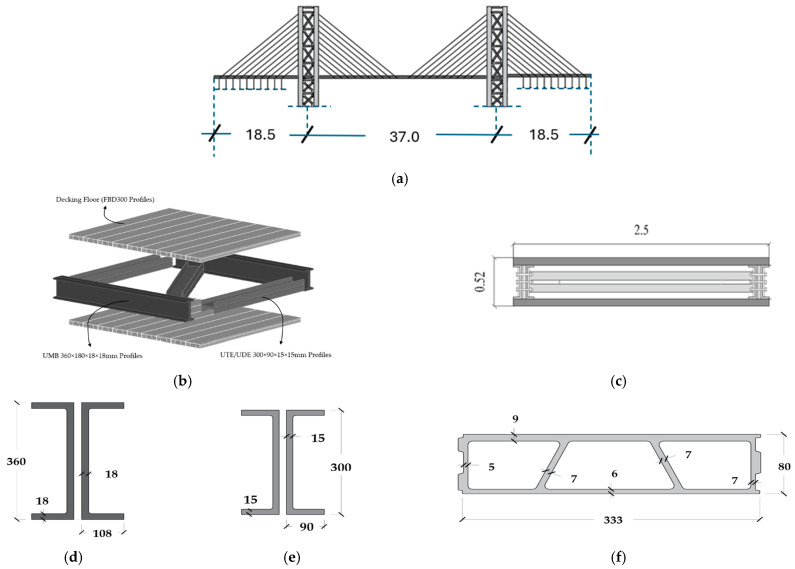
Salerno footbridge (Italy, 2020): (**a**) side view (dimensions provided in meters [m]); (**b**) 3D visualization of the deck system; (**c**) cross-sectional representation of the deck system (dimensions provided in meters [m]); (**d**) FRP profiles implemented for main girders (dimensions provided in millimeters [mm]); (**e**) FRP profiles implemented for cross-bars (dimensions provided in millimeters [mm]); (**f**) FRP profiles implemented for deck (dimensions provided in millimeters [mm]).

**Figure 9:**
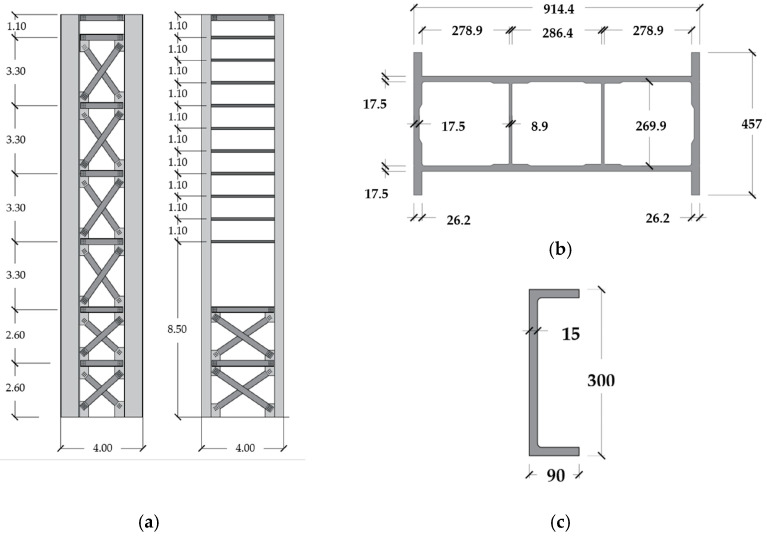
Pylons designed for Salerno bridge: (**a**) general view (dimensions provided in meters [m]); (**b**) FRP profiles implemented for the main construction of pylons (dimensions provided in millimeters [mm]); (**c**) FRP profiles implemented for the stiffening girders of pylons (dimensions provided in millimeters [mm]).

Nisticò and coauthors [3] introduced the preliminary design of a cable-stayed bridge, appropriately named the Salerno Bridge. The proposed site for the bridge is situated within the University of Salerno (Italy) campus. The bridge (Figure 8 and Figure 9) incorporates the following components: (1) PCFRP cables (ϕ 12 [mm]), fastened by split wedge anchorages [37,38]; (2) GFRP sandwiches decks (Figure 8b,c), comprising an intermediate system with a) pairs of U-shaped longitudinal (Figure 8d) and diagonal/transversal elements (Figure 8e) whose material has undergone experimental and numerical studies as documented in [39,40,41]; (b) top and bottom panels (Figure 8f) (3) pylons (Figure 9a) assembled through a) four vertical Double Web Beam (Figure 9b); (b) U-shaped with the section geometry reported in Figure 9c. The mechanical properties of the adopted materials are detailed in Table 10. One of the primary design goals was to limit the deck acceleration resulting from pedestrian actions. Consequently, the target frequency were set as follows: (1) for horizonal modes, 2.5 [Hz] which, according to Euro Code [14], is the lower threshold to avoid specific studies; (2) for vertical modes, approximately ≈ 3.5 [Hz] which, among the frequency lower than 5.0 [Hz], is the frequency at which the factor reported in Figure 2 has a minimum for jogging and almost a minimum for walking.

Within this framework, a comprehensive investigation will be undertaken on the Salerno Bridge, examining a three-span design intended to also serve as a railway overpass.

Two variants of the three-span cable-stayed bridge have been examined, including configurations with tie rods (Variant A) and without tie rods (Variant B).

**Figure 10:**
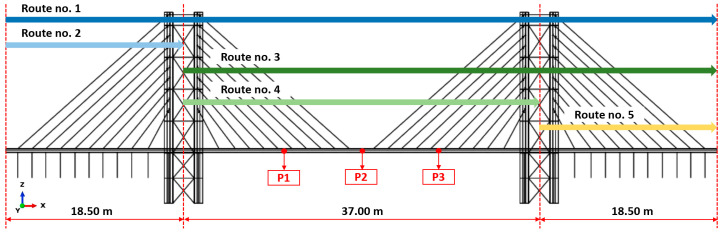
Salerno footbridge: three-span solution including tie rods. Route variants considered for user dynamic performance (dimensions provided in [m]).

The methodology employed for evaluating comfort is derived from the approach utilized in studying the Aberfeldy footbridge, as detailed in [28]. This assessment encompasses vibrations induced by both pedestrians and trains. Pedestrians are expected to follow one of the five designated routes (Figure 10), with seven combinations considered, as outlined in Table 11. Pedestrians in the group are assumed to walk or run under continuous flow conditions. The simulation of train travel involved applying the recorded acceleration time history at the base of the pylons.

**Figure 11:**
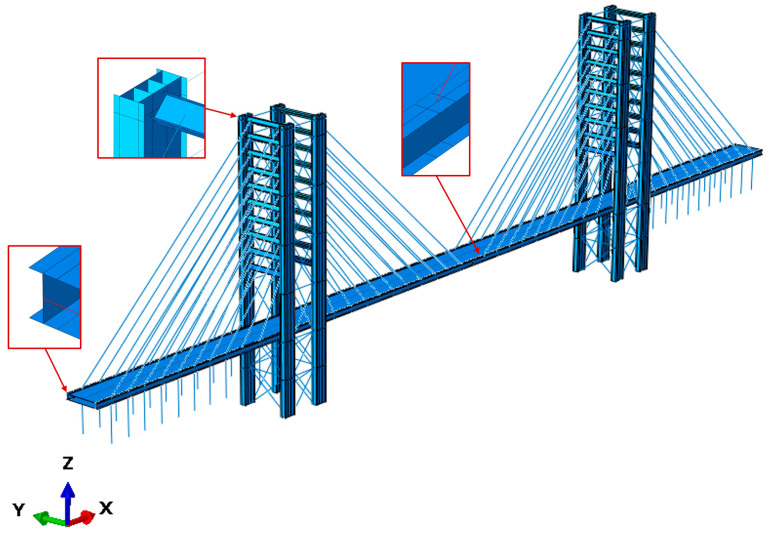
FE model serving as a numerical representation of the Salerno footbridge.

The finite element method (FEM) model of the footbridge (Figure 11), implemented using ABAQUS/Standard [42], comprises 151,051 linear S4R and T3D2 elements. Truss elements were used for the discretization of cables, utilizing an equivalent Young’s modulus. Additionally, shell orthotropic elements, applying lamina theory, were used to simulate the pylon and deck elements.

### 3.1. Mode Shapes and Frequencies

**Figure 12:**
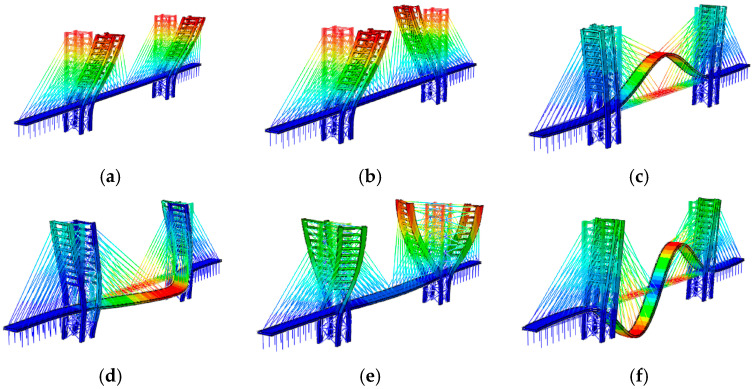
The mode shapes and frequencies obtained for Variant A of the Salerno footbridge: (**a**) $f_{1}=2.11\;[Hz]$, (**b**) $f_{2}=2.12\;[Hz]$, (**c**) $f_{3}=4.04\;[Hz]$, (**d**) $f_{4}=5.27\;[Hz]$, (**e**) $f_{5}=5.77\;[Hz]$, (**f**) $f_{6}=6.06\;[Hz]$.

**Figure 13:**
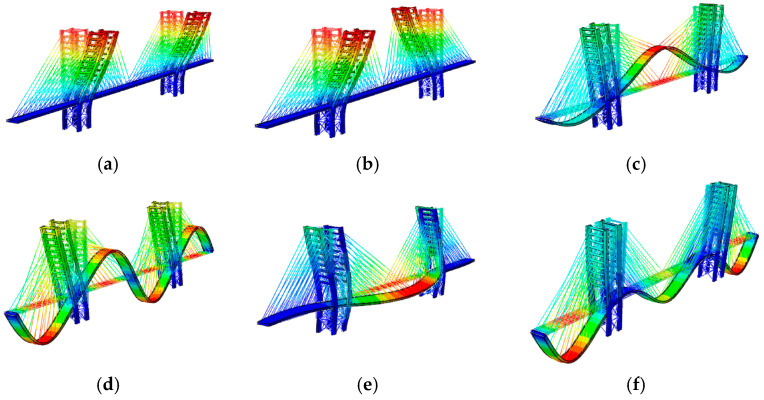
The mode shapes and frequencies obtained for Variant B of the Salerno footbridge. B: (**a**) $f_{1}=2.10\;[Hz]$, (**b**) $f_{2}=2.11\;[Hz]$, (**c**) $f_{3}=3.64\;[Hz]$, (**d**) $f_{4}=4.92\;[Hz]$, (**e**) $f_{5}=5.13\;[Hz]$, (**f**) $f_{6}=6.48\;[Hz]$.

For both variants, the following observations can be made by combining mode shapes (Figure 12 and Figure 13) and frequencies (Table 12). Modes 1 and 2 primarily concern the excitations of the antennas along the transverse and longitudinal directions of the bridge. Additionally, mode 4 (Variant A) and 5 (Variant B), related to the bridge deck, are lateral, and their frequencies, close to 5 Hz, are greater than 2.5 Hz. According to the Euro Code [14], this implies that comfort problems can be excluded. Furthermore, modes 3 of both variants involve the vertical direction of the deck. Their frequencies, 4.04 and 3.64, are lower than 5 Hz. While the risk of resonance cannot be entirely excluded according to the Eurocode [14], it is considered low according to SÉTRA [13], as reported in Table 6.

### 3.2. Pedestrian-Induced Vibrations

As discussed in Section 2.2, various models can be employed to simulate walking and running pedestrians. Thus, for the sake of comparison, the model originally proposed by Bachmann and co-authors was initially adopted. Equation (1), comprising three terms, was utilized to simulate walking and running. In Table 11, the set of all investigated passages of one user is presented.

Subsequently, running and walking conditions were simulated using the half-sine model proposed in Equation (2), SÉTRA [13] in Equations (6) and (7), and British Standard [16] in Equations (8) and (9). All simulations pertained to vertical excitation, given the high value (approximately 5 [Hz]) of lateral deck modes.

#### 3.2.1. Walking Condition

The mathematical model for walking cases expressed by Equation (1) has been evaluated using the parameter values [20] outlined in Table 2: (1) the selected frequencies are incorporated into the first term of the series, and (2) the series encompasses two additional higher frequencies. Some of the selected frequencies align with the vertical modes of the deck, while others are deliberately tuned to replicate realistic frequencies for both walkers and runners.

Table 13 presents the selected cases and their corresponding peak accelerations, considering both Variant A (AV) and Variant B (BV) of the bridge configuration, as illustrated in Figure 10 and Table 11. The selection of pacing frequency was made based on (1) typical pedestrian gait and (2) natural frequencies and modes of the structure. The assumed step length is 0.75 [m], and the pedestrian is assumed to walk along Route 1. Furthermore, the most critical scenarios (AV.2.1, BV.2.1) were chosen to explore the impact of damping and path dependence, with the results documented in Table 14 and Table 15, respectively. Seven scenarios were considered for each case.

From the analysis, it can be concluded that under the assumption of a pedestrian traveling along Route 1, the most unfavorable conditions arise. When a damping value of 1.5% is considered, the highest values of the peak acceleration values (PAVs) are 0.26 [m/s^2^] and 0.18 [m/s^2^], respectively for Variant A and B. If the damping value is reduced to 0.5%, the PAV increases to 0.55 [m/s^2^]. Notably, all aforementioned PAVs remain below the threshold of 0.7 [m/s^2^] recommended in the Euro Code [14].

Additionally, considering SÉTRA [13] and assuming a damping of 0.5%, the comfort level can be regarded as maximum for Variant A and nearly maximum for Variant A (refer to Table 15). A marginal increase in damping is adequate for Variant B to achieve the maximum comfort level.

The critical load scenarios were analyzed according to British Standard [16], using Equation (8), where N is assumed based on the footbridge class (see Table 3). The two extreme cases, N = 2 and 16, are considered. The reference amplitude of the applied fluctuating force $F_{0}$ is 0.28 [kN], $k\left(f_{v}\right)$ is 0.38 (in accordance with the function depicted in Figure 2, and γ is 0.0.34 (Figure 4). The analysis was conducted for L = 37 [m], assuming a logarithmic decrement of 9%, resulting in a critical damping of 1.5%. In Table 16, results obtained from this approach are summarized.

#### 3.2.2. Running Condition

For the running cases, the half-sine model, as described by Equation (2), has been assessed using the parameter values provided in Table 2. The used step length is 1.5 [m], and the user is assumed to run along Route 1. Table 17 displays the chosen scenarios along with their respective maximum accelerations, encompassing both Variant A (AV) and Variant B (BV) of the bridge design.

#### 3.2.3. Crowded Conditions: SÉTRA [13] and British Standard [16]

In crowded conditions, the increase in mass affects the frequency. So, considering a density of 1.0 [person/m^2^] and supposing the pedestrian mass equal to 70 [kg], it follows that (1) for Variant A, the frequency under consideration, which is initially 4.04 [Hz], decreases to 3.70 [Hz]; and (2) for Variant B, where two modes are under investigation, the frequencies decrease from 3.64 [Hz] to 3.36 [Hz] and from 4.92 [Hz] to 4.57 [Hz], respectively.

SÉTRA [13] refers to Equation (6), considering the frequency dependent function ($\psi_{1}$) reported in Figure 1a that excludes the frequencies greater than 2.6 [Hz], assuming for them $\psi_{1}=0$. Consequently, the bridge frequencies of 3.7 [Hz] (Variant A), 3.36, and 4.57 [Hz] (Variant B) must be considered only for the second term (see Equation (7)), that includes the $\psi_{2}$ function reported in Figure 1b. Finally, to evaluate the acting force for unit of area, Equation (12) holds, (1) assuming a density (ρ) of 1 [person/m^2^], and (2) evaluating the equivalent number of pedestrians as reported in Equation (3), where N is the real number of pedestrians walking in the global area (A = 148 [m^2^]) of the bridge.

(12) $$w\left(t\right)=\frac{0.13\left(kN\right)}{A}\cdot \sqrt{N}{\cdot \psi }_{2}\cdot cos(2\pi f_{1}t)\;$$

British Standard [16] refers to Equation (8) in normal conditions and to Equation (9) for crowded conditions. Furthermore, in the case of walking conditions, a velocity of 1.7 [m/s] is defined. Considering a pedestrian step of 0.8 [m], it results in a load frequency equal to 1.0 [Hz], which is sufficiently far from the bridge frequencies ranging between 3.36 and 4.04 [Hz]. For jogging, a velocity of 3.0 [m/s] needs to be assumed. Considering a step length of 1.6 [m], the load frequency (≈1.0 [Hz]) is also far from the considered frequencies for both walking and running cases.

With these premises, it has been decided, in any case, to induce a flow of pedestrians, in crowded conditions, to walk in resonance with the bridge frequencies.

Equation (9) has been modified, as shown in Equation (13), since in crowd conditions, (1) independently on the span length, γ is linearly dependent (Figure 4) on the logarithmic decrement (≈2πξ) and consequently linerly dependent on critical damping (γ=7.0·ξ); (2) $F_{0}$ has been assumed to be equal to 0.28 [kN]; (3) for $k\left(f_{v}\right),$ a value equal to 0.38 has been assumed, according to Figure 2; and (4) λ=0.634, assuming (Figure 3) $S=S_{eff}$.

(13) $$w(t)=\frac{0.63\;(kN)}{A}\sqrt{\xi \cdot N}\cdot sin\left(2\pi \cdot f\cdot t\right)$$

**Figure 14:**
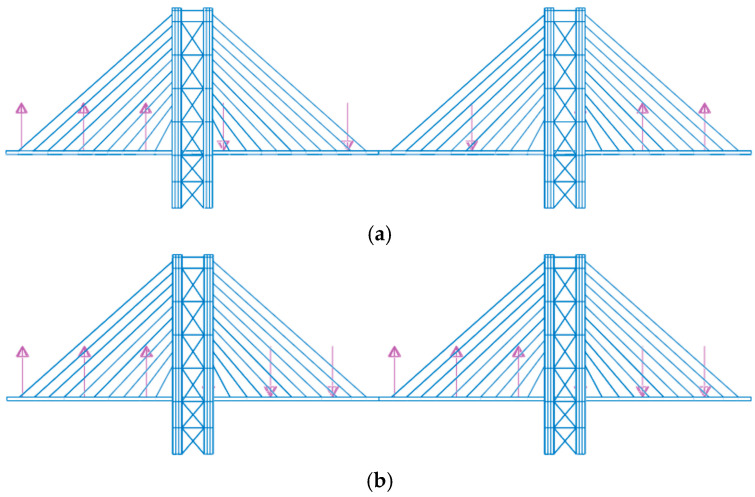
Applied loads for crowd condition assessment that induced extreme accelerations for (**a**) the first vertical mode of Variant A and B, and (**b**) the second vertical mode of Variant B.

Both Equations (12) and (13) have been considered to evaluate the comfort. To achieve the worst condition: (1) the direction of pedestrian acting forces has been defined, as reported in Figure 14, given the mode shapes reported in Figure 12 (Variant A) and Figure 13 (Variant B); (2) the steady state condition was referred to.

**Figure 15:**
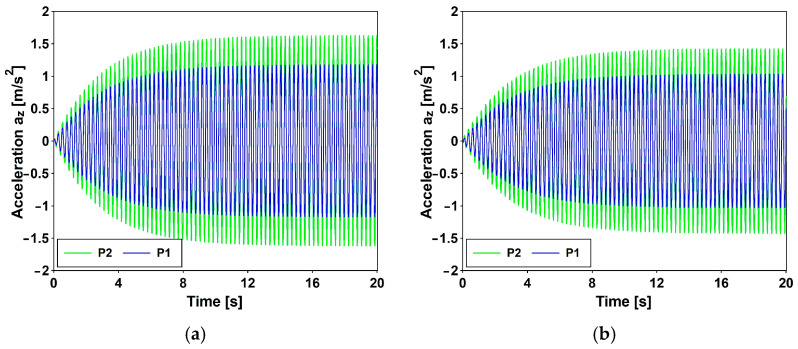
Time history of accelerations. Variant A: crowd conditions evaluated based on (**a**) British Standard [16] and (**b**) SETRA [13].

The resulting peak acceleration values (PAVs) are documented in Table 18 and Figure 15, revealing a notable discrepancy from the desired standards. Notably, the highest recorded values, assuming ξ=0.15, are more than twice as high as both the Eurocode [14] and British Standards [16] thresholds. According to SÉTRA [13], the comfort level can only be classified as minimal, since, referring to Table 1, all the PAVs are greater than 1.0 [m/s^2^]. Recalling that induced resonance is not a realistic scenario, the authors believe that a viable solution can still be pursued by designing the system to enhance structural damping, potentially through the integration of dampers or tuned mass dampers (TMD).

### 3.3. Traffic-Induced Actions

**Figure 16:**
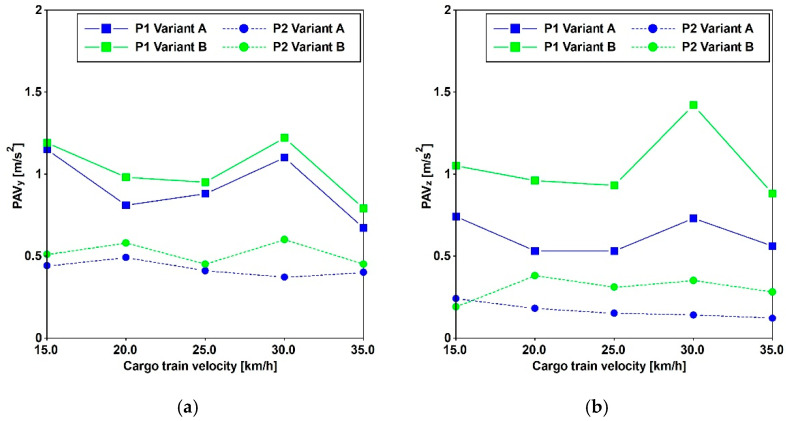
Comparison between Variant A and Variant B. PAVs for cargo train passages in the Y (**a**) and Z (**b**) directions.

**Figure 17:**
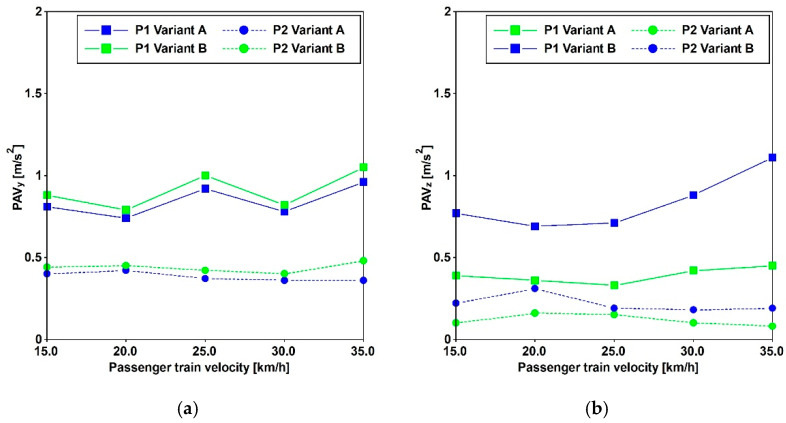
Comparison between Variant A and Variant B. PAVs for passenger train passages in the Y (**a**) and Z (**b**) directions.

Train travel simulations were conducted by imposing the recorded accelerations, as introduced in Section 2.1, at the base of the antennas. Both cargo and passenger trains were subjected to five constant velocities, and the time histories were recorded at various distances. The results of these analyses, presented in Figure 16 and Figure 17, depict the peak accelerations (PAVs) at three different positions. These results reveal that, at the central point of the main span, the thresholds commonly adopted for pedestrian-induced vibrations are not met. The highest values are associated with velocities of 30 for the cargo train and 35 [km/h] for the passenger train.

In the horizontal direction, the PAVs consistently exceed the Euro Code [14] threshold of 0.2 [m/s^2^], even when the horizontal frequencies exceed 2.5 [Hz]. Regarding vertical acceleration, Variant A surpasses 0.7 [m/s^2^], while Variant B meets this threshold for the passenger train and is close to meeting it for the cargo train.

Furthermore, in accordance with SÉTRA [13], the comfort levels can be evaluated based on the criteria reported in Table 5. It is minimum for horizontal accelerations, applicable to both variants, and for vertical acceleration when considering Variant A. However, for Variant B, the comfort level is maximum for the passenger train and medium for the cargo train.

**Figure 18:**
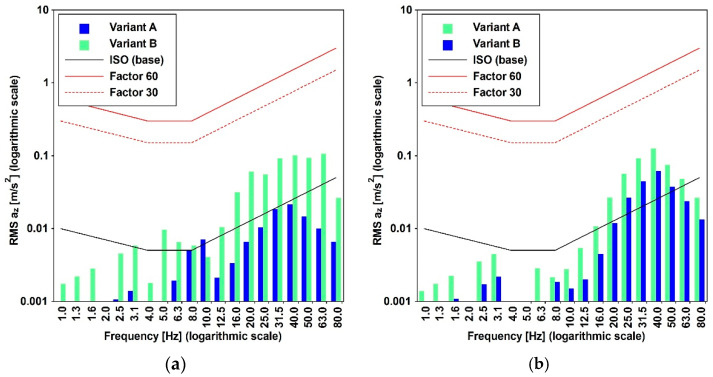
Variant A vs. B. Comfort criteria assessment for foot-to-head direction based on ISO [15] standard. Passage of: (**a**) cargo train (speed 30 [km/h]); (**b**) passenger train (speed 35 [km/h]). RMS values at the middle span of the structure (B control point).

**Figure 19:**
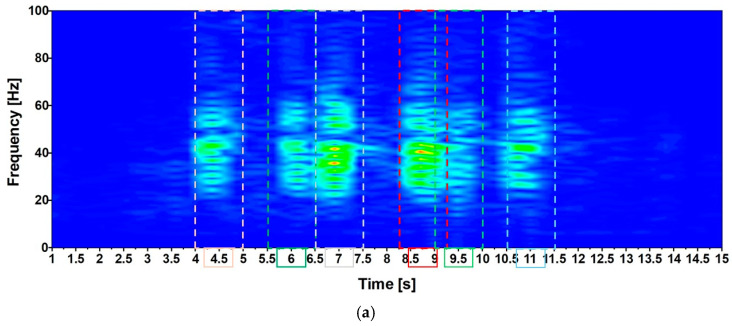

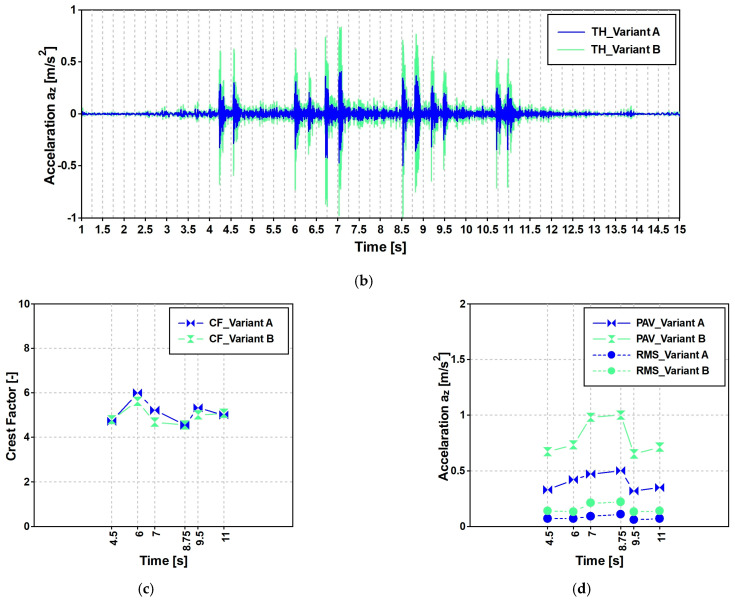
Analysis of the acquired signal. Variant A vs. B. (**a**) Example of acceleration time history; (**b**) crest factors; (**c**) PAV and (**d**) RMS values.

However, considering the high frequency characterizing the input signal, as denoted in Figure 6, it seems more appropriate to apply the methodology outlined in ISO [15], as applied and documented in Figure 18, by employing the root mean square (RMS) method (Equation (11)). The applicability of the method is supported by the value (lower than 9) of the crest factor (PAV/RMS) reported in Figure 19c.

The RMS has been evaluated, considering the six intervals reported in Figure 19a that refer to the time history reported in Figure 19b. These intervals have been selected assuming ΔT = 1.0 [sec] in Equation (1), and for each interval, the RMS (Figure 19c) has been evaluated. The highest values of the RMS are ≈ 0.1 for Variant A and 0.2 for Variant B. These values are attained in a frequency range between 7 and 8.75 [Hz], where the RMS (Figure 7b) assumes values of ≈0.23, having considered a scale factor equal to 30 that concerns the standing condition. It follows that the limitations are respected (see Figure 18).

## 4. Conclusions

The design of pedestrian bridges is significantly influenced by pedestrian-induced actions, and regulatory recommendations primarily address two main aspects: pedestrian action modelling and suggestions to limit induced accelerations. Frequency thresholds are defined to avoid specific evaluations, and acceleration limits are introduced to assess structural performance. Eurocode [14] and British Standard [16] set mandatory acceleration thresholds, while SÉTRA [13] classifies comfort levels based on a set of acceleration levels. ISO [15], not specifically designed for bridges, introduces the RMS, an integral index of accelerations.

Models adopted for pedestrian walking and running are often conservative, especially in crowd conditions. Frequency limitations and acceleration thresholds recommended may not guarantee optimal performance. Consequently, designers need to anticipate modifications to be installed after construction in case of observed deficiencies.

In this context, a prototype of a fully FRP cable-stayed bridge was proposed [3] by Nisticò and coauthors. The primary challenges were wind and pedestrian effects, with the latter being the focus of this work. Initially, adopting the Bachman proposed pedestrian model [25], the bridge’s performance was investigated. The results indicated good performance, surpassing other cable-stayed and arch FRP pedestrian bridges.

Subsequently, evaluations were conducted based on pedestrian group scenarios using the pedestrian model suggested by SÉTRA [13] and British Standard [16]. Defining an equivalent group of pedestrians supposed to be synchronized required statistical data acquired by SÉTRA [13]. The results highlighted that in cases of walking and running in normal conditions, the performance aligns with standard recommendations if the critical damping is assumed to be equal to 1.5%.

However, in crowded conditions, it has been shown that the step frequency of pedestrians cannot be in resonance with the structural frequencies. Nevertheless, by forcing pedestrians in crowded dense conditions to be in resonance with the structural frequencies, a notable discrepancy from the desired standards was observed. Viable solutions could be pursued by designing a system to enhance structural damping.

Finally, the study extended to evaluating the comfort induced by cargo and passenger trains. Following the methodology for comfort evaluation, the analyses were performed based on ground acceleration histories from Drygala et al. [29]. The performance was deemed satisfactory.

In conclusion, the proposed bridge’s performance can be considered adequate according to international standards, even if, in crowded conditions, more specific studies will be required. The evaluation assumed a critical damping up to 1.5%, aligning with the mean value suggested in [43], which is considered realistic. Critical damping for FRP bridges is lower than timber structures [27] but surpasses that of steel, concrete, and steel-composite footbridges, reaching, in some cases [8], 3%. Further studies will focus on the effects of running in crowded conditions, including marathons, wind-induced actions, and the consequent design of dampers or tuned mass dampers to be implemented in case of experienced non-performing structural behavior.

## Figures and Tables

**Table 1:** Frequency, velocity, and step length range for walking and running.

Type of Motion	Frequency $f_{u}$ [Hz]	Velocity $v_{u}$ [m/s]	Stride Length $l_{u}$ [m]
Walking	2.00	1.20	0.60
Fast walking	2.50	2.50	1.00
Slow running	2.80	3.50	1.25
Fast running	≈4.00	7.00	1.70

**Table 2:** Human-induced dynamic load (walking and running). Equations (1) and (2), parameters according to [20,21,22,25].

**Equation (1) (Walking and Running)**								
**Motion**	**Reference**	G[kN]	$\alpha_{1}$	$\alpha_{2}$	$\alpha_{3}$	$\varphi_{1}$	$\varphi_{2}$	$\varphi_{3}$
Walking	[25]	0.80	0.4	0.10	0.10	0	π/2	π/2
	[20]	0.70	0.37	0.10	0.12	0	π/2	π/2
Running	[25]	0.80	1.6	0.70	0.2	0	0	0
	[20]	0.70	1.45	0.15	0.05	0	0	0
**Equation (2) (Running) [21,22]**								
**T [s]**	G[kN]	$t_{c}$ **[s]**		k		$A_{r}$		**T [s]**
0.37	0.70	0.23		0.62		2.53		0.37
0.39	0.70	0.29		0.73		2.15		2.40
0.45	0.70	0.31		0.68		2.31		3.20

**Table 3:** British Standard [16]. Human-induced dynamic load: walking (W) and jogging (J). Parameter values for Equation (3): N—pedestrian number; A—deck area; ρ—crowd density [persons/m^2^] (with a maximum of 1.0 for the pedestrian velocity reduction in crowd condition).

Type of Motion	$F_{0}$ [kN]	Speed [m/s]	N (Pedestrian Number) and ρ (Crowd Density)			
			Rural (A)	Sub-Urban (B)	School (C)	Stadium (D)
Normal W	0.28	1.3	N = 2	N = 4	N = 8	N = 16
Normal J	0.91	3.0	N = 0	N = 1	N = 2	N = 4
Crowd W and J	0.28	-	0	ρ=0.4 N=0.4 A	ρ=0.8 N=0.8 A	ρ=1.5 N=1.5 A

**Table 4:** PGAs for different train velocities for the lateral (x, y) and vertical (z) directions.

Type of Train	Velocity [km/h]	$\mathrm{Peak}\;\mathrm{Ground}\;\mathrm{Accelerations}\;PGA\;[\frac{m}{s^{2}}]$					
		P1			P2		
		*a_x_*	*a_y_*	*a_z_*	*a_x_*	*a_y_*	*a_z_*
Cargo	15.00	0.74	0.79	0.73	0.14	0.40	0.26
	20.00	0.65	0.74	0.60	0.12	0.40	0.21
	25.00	0.73	0.69	0.61	0.12	0.41	0.19
	30.00	0.87	0.72	0.81	0.13	0.53	0.30
	35.00	0.65	0.55	0.61	0.14	0.34	0.24
Passenger	15.00	0.62	0.66	0.55	0.11	0.31	0.17
	20.00	0.72	0.65	0.46	0.10	0.31	0.20
	25.00	0.86	0.78	0.64	0.11	0.33	0.15
	30.00	0.72	0.70	0.57	0.11	0.28	0.11
	35.00	0.73	0.83	0.64	0.10	0.33	0.11

**Table 5:** SÉTRA [13]: acceleration limits $\left[\frac{m}{s^{2}}\right]$ vs. ranges for the comfort classification.

Comfort Level	$\mathrm{Ranges}\;\mathrm{of}\;\mathrm{Comfort}\;[\frac{m}{s^{2}}]$	
	Vertical	Horizontal
Maximum	0.0–0.5	0.00–0.15
Mean	0.5–1.0	0.15–0.30
Minimum	1.0–2.5	0.30–0.80
Uncomfortable	>2.5	>0.8

**Table 6:** The resonance risk levels and corresponding ranges of natural frequencies [13].

Risk of Resonance	Frequency Ranges [Hz]	
	Vertical	Horizontal
Maximum	1.7–2.1	0.5–1.1
Medium	1.0–1.7; 2.1–2.6	0.3–0.5; 1.1–1.3
Low	2.6–5.0	1.3–2.5
Negligible	0–1.0; >5.0	0–0.3; >2.5

**Table 7:** British Standard [16]: recommended values for response modifiers.

Response Modifier	Recommended Value and Description * Multiplying Factor
Site usage factor $k_{1}$	0.6—HSR and PR for hospital
	0.8—PR for school
	0.8—HUR and PR for sport stadium
	1.0—MUC
	1.3—SC
	1.6—RE
Route redundancy factor $k_{2}$	0.7—SMA
	1.0—PR
	1.3—ARRA
Structure heigh factor $k_{3}$	0.7—greater than 8 [m]
	1.0–4 [m] to 8 [m]
	1.1—less than 4 [m]

* HSR—Highly Sensitive Route, PR—Primary Route, HUR—High Usage Routes, MUC—Major Urban Center, SC—Suburban Crossing, RE—Rural Environment, SMA—Sole Means Access, ARRA—Alternative Routes Readily Available.

**Table 8:** ISO [15]: Multiplying factors to adopt to scale the function reported in Figure 1.

Direction of Vibration	Pedestrian Scenario	Multiplying Factor
Vertical	Standing	30
Vertical	Walking	60
Horizontal	Standing or walking	60

**Table 9:** Evaluation of main pedestrian bridge frequencies [Hz].

Bridge	Typology	Vertical	Horizontal	Torsional
Aberfeldy [4]	Cable-Stayed	1.52	0.93	-
Kolding [5]	Cable-Stayed	4.3	-	6.59
Leida [8]	Arch	2.75	-	-
Wilcott [6]	Suspension	0.96	1.00	-
Prato [10]	Truss	7.5	5.8	-
Blackpool [29]	U-Shaped	9.20	5.71	-

**Table 10:** Salerno Bridge: material mechanical properties. E—Young Modulus [GPa]; G—shear modulus [MPa]; f—axial strength [MPa]; τ—shear strength [MPa]. Tension (+), compression (−).

Element	*E*_0_	*E*_90_	*G*	*f*_0_	*f*_90_	*τ*
U-shaped	24.0	10.0	3.0	+240; −240	45.0	20.0
DWB	39.0	10.0	3.0	+206; −206	45.0	20.0
Panel	27.0	14.0	3.0	+350; −205	100.0	20.0
CFRP cables	164.0	N.A.	N.A.	2275.0	N.A.	N.A.

**Table 11:** Selected routes and the corresponding number of pedestrians.

Paths	No of Pedestrians
1—Route no. 1	1
2—Route no. 2	1
3—Route no. 3	1
4—Route no. 4	1
5—Route no. 2 and 5	1 + 1
6—Route no. 2, 4, and 5, in phase	1 + 1 + 1
7—Route no. 2, 4, and 5, counterphase	1 + 1 + 1

**Table 12:** Frequencies of the Salerno footbridge for Variant A and Variant B.

Mode i [−]	Variant A		Variant B	
	$f_{i}\;[Hz]$	Direction	$f_{i}\;[Hz]$	Direction
1	2.11	H-Piers	2.10	H-Piers
2	2.12	H-Piers	2.11	H-Piers
3	4.04	V-Deck	3.64	V-Deck
4	5.27	H-Deck	4.92	V-Deck
5	5.77	V-Deck	5.13	H-Deck
6	6.06	V-Deck	6.48	V-Deck

**Table 13:** Dynamic response of the footbridge variants A (AV) and B (BV). PAV values corresponding to one pedestrian passage (PAVs). Assumed damping ratio: 1.5%.

No. of Load Variant	FE Natural Frequency of Footbridge [Hz]	Frequency of Loading [Hz]	$PAVs\;[\frac{m}{s^{2}}]$		
			P1	P2	P3
AV.1.1	-	1.70	0.04	0.05	0.04
AV.1.2	-	2.00	0.23	0.19	0.22
AV.1.3	-	2.30	0.06	0.09	0.06
BV.1.1	-	1.70	0.08	0.05	0.07
BV.1.2	-	2.00	0.04	0.05	0.03
BV.1.3	-	2.30	0.05	0.05	0.05
AV.2.1	4.04	2.02	0.26	0.23	0.25
BV.2.1	3.64	1.82	0.12	0.18	0.12

**Table 14:** PAVs at P1 and P2 points vs. damping ratio.

Variant	$\mathrm{PAVs}\;[\frac{m}{s^{2}}]$ vs. Damping Ratio [%]											
	0.5		1.0		1.5		2.0		2.5		3.0	
	P1	P2	P1	P2	P1	P2	P1	P2	P1	P2	P1	P2
A V.2.1	0.55	0.45	0.35	0.30	0.26	0.23	0.21	0.19	0.17	0.16	0.15	0.14
B V.2.1	0.28	0.36	0.17	0.23	0.12	0.18	0.10	0.14	0.09	0.12	0.08	0.10

**Table 15:** PAVs at P1 and P2 points vs. paths (damping values equal to 1.5%).

Scenario	PAVs $\left[\frac{m}{s^{2}}\right]$					
	AV.2.1			BV.2.1		
	P1	P2	P3	P1	P2	P3
No. of ped.: 1; Rt. no.: 1	0.26	0.23	0.25	0.12	0.18	0.12
No. of ped.: 1; Rt. no.: 2	0.02	0.02	0.02	0.09	0.06	0.06
No. of ped.: 1; Rt. no.: 3	0.26	0.23	0.25	0.12	0.18	0.12
No. of ped.: 1; Rt. no.: 4	0.26	0.23	0.25	0.12	0.18	0.12
No. of ped.: 2; Rt. no.: 2 and 5	0.02	0.02	0.02	0.08	0.13	0.08
No. of ped.: 3; Rt. no.: 2, 4, and 5; in-phase	0.27	0.24	0.26	0.12	0.15	0.12
No. of ped.: 3; Rt. no.: (2 and 5), 4 in counter-phase	0.25	0.22	0.24	0.18	0.25	0.16

**Table 16:** BSI [16]: PAVs at P1 and P2 points vs. for footbridge class.

Footbridge Class	N	$PAVs\;[\frac{m}{s^{2}}]$ vs. Load Variant (Frequency [Hz])					
		A V.2.1			B V.2.1		
		P1	P2	P3	P1	P2	P3
A	2	0.19	0.29	0.19	0.16	0.24	0.16
D	16	0.36	0.55	0.36	0.30	0.46	0.30

**Table 17:** Dynamic response of the footbridge for the one runner passage (PAVs).

No. of Load Variant	FE Natural Frequency of Footbridge [Hz]	Frequency of Loading [Hz]	$PAVs\;[\frac{m}{s^{2}}]$		
			P1	P2	P3
A V.1.4	-	2.40	0.10	0.10	0.09
A V.1.5	-	3.20	0.20	0.23	0.19
B V.1.4	-	2.40	0.13	0.12	0.12
B V.1.5	-	3.20	0.18	0.28	0.16
A V.2.2	6.06	3.03	0.38	0.23	0.35
B V.2.2	4.92	2.47	0.23	0.11	0.22
B V.2.3	6.48	3.24	0.36	0.22	0.33

**Table 18:** PAVs for crowd conditions based on SÉTRA [13] and BSI [16] for damping ratio 1.5%.

Standard	$PAVs\;[\frac{m}{s^{2}}]$ vs. Load Variant (Frequency [Hz])								
	AV—f = 3.70 [Hz]			BV—f = 3.36 [Hz]			BV—F = 4.57 [Hz]		
	P1	P2	P3	P1	P2	P3	P1	P2	P3
SÉTRA [13]	1.03	1.43	1.03	1.19	1.59	1.19	1.09	0.05	1.09
BS [16]	1.16	1.57	1.16	1.38	1.85	1.38	1.39	0.07	1.39

## Data Availability

Data are contained within the article.
